# A Novel Thiazolyl Schiff Base: Antibacterial and Antifungal Effects and *In Vitro* Oxidative Stress Modulation on Human Endothelial Cells

**DOI:** 10.1155/2019/1607903

**Published:** 2019-10-10

**Authors:** Cristian Cezar Login, Ioana Bâldea, Brînduşa Tiperciuc, Daniela Benedec, Dan Cristian Vodnar, Nicoleta Decea, Şoimiţa Suciu

**Affiliations:** ^1^Department of Physiology, Iuliu Hațieganu University of Medicine and Pharmacy, 1 Clinicilor Street, 400006 Cluj-Napoca, Romania; ^2^Department of Pharmaceutical Chemistry, Iuliu Hațieganu University of Medicine and Pharmacy, 41 Victor Babeș Street, 400012 Cluj-Napoca, Romania; ^3^Department of Pharmacognosy, Iuliu Hațieganu University of Medicine and Pharmacy, 12 Ion Creangă Street, 400010 Cluj-Napoca, Romania; ^4^Department of Food Science, University of Agricultural Sciences and Veterinary Medicine, 3-5 Mănăștur Street, 400372 Cluj-Napoca, Romania

## Abstract

Schiff bases (SBs) are chemical compounds displaying a significant pharmacological potential. They are able to modulate the activity of many enzymes involved in metabolism and are found among antibacterial, antifungal, anti-inflammatory, antioxidant, and antiproliferative drugs. A new thiazolyl-triazole SB was obtained and characterized by elemental and spectral analysis. The antibacterial and antifungal ability of the SB was evaluated against Gram-positive and Gram-negative bacteria and against three *Candida* strains. SB showed good antibacterial activity against *L. monocytogenes* and *P. aeruginosa*; it was two times more active than ciprofloxacin. Anti-*Candida* activity was twofold higher compared with that of fluconazole. The effect of the SB on cell viability was evaluated by colorimetric measurement on cell cultures exposed to various SB concentrations. The ability of the SB to modulate oxidative stress was assessed by measuring MDA, TNF-*α*, SOD1, COX2, and NOS2 levels *in vitro*, using human endothelial cell cultures exposed to a glucose-enriched medium. SB did not change the morphology of the cells. Experimental findings indicate that the newly synthetized Schiff base has antibacterial activity, especially on the Gram-negative *P. aeruginosa*, and antifungal activity. SB also showed antioxidant and anti-inflammatory activities.

## 1. Introduction

Aerobic organisms have antioxidant defense systems against reactive oxygen species- (ROS-) induced damage produced in various stress conditions. ROS are also involved in the innate immune system and have an important role in the inflammatory response; they attract cells, by chemotaxis, to the inflammation site. Nitric oxide (NO) is another important intracellular and intercellular signaling molecule involved in the regulation of multiple physiological and pathophysiological mechanisms. It acts as a biological modulator. NO is able to regulate vascular tone and can function as a host defense effector. Also, it can act as a cytotoxic agent in inflammatory disorders. NO synthase (NOS) enzyme family catalyzes NO production. Inhibition of inducible NOS (iNOS) might be beneficial in the course of treatment of certain inflammatory diseases [1]. The reactions between NO and ROS, such as superoxide radicals (O_2_^·−^), lead to the production of a potent prooxidant radical (peroxynitrite), thus inducing endothelial and mitochondrial dysfunction. The major cellular defense against peroxide and peroxynitrite radicals are the superoxide dismutases (SODs) that catalyzes the transformation of peroxide radicals into hydrogen peroxide (H_2_O_2_), which is further transformed by catalase into water and molecular oxygen. Also, SODs play an important role in preventing peroxynitrite formation [2]. All isoforms have in their catalytic site a transition metal, such as copper and manganese [3].

Recent studies showed that exogenous NO, produced by bacterial NOS, protects Gram-positive and Gram-negative bacteria (*Pseudomonas aeruginosa*, *Staphylococcus aureus*, etc.) against oxidative stress and increases bacterial resistance to a broad spectrum of antibiotics [4]. Fungal resistance to antimycotic treatment is one of the consequences of the emergence of resistant strains, but more and more, in the last years, fungal resistance is due to the capacity of fungal strains to form biofilms, which are considered critical in invasive fungal infections, associated with high mortality. Certain studies showed that only a few antimycotics are effective against fungal biofilms. All of them have the capacity to induce ROS formation in fungal biofilm cells [5]. In this context, finding bioactive substances capable to reduce NO synthesis in bacteria or able to induce ROS synthesis in fungal biofilms could represent new directions in the development of new antimicrobial drugs. Thiazoles, triazoles, and their derivatives are found among antibacterial and anti-inflammatory drugs [6–9].

Schiff bases (SBs) are chemical structures that have a significant pharmacological potential. SBs contain an azomethine group obtained through the condensation of primary amines with carbonyl compounds [10]. The pharmacophore potential of this group is due to their ability to form complex compounds with bivalent and trivalent metals located in the active center of numerous enzymes involved in metabolic reactions. The relationship between a chemical structure and biological activity (SAR) underlines the importance of the azomethine group for the synthesis of new compounds with antibacterial, antifungal, and even antitumor activities [11–13].

Multiple studies showed the ability of SBs to act as antiproliferative and antitumoral agents [14–16]. The azomethine pharmacophore is used in developing new bioactive molecules [17]. The discovery of selective cytotoxic drugs influenced oncological therapy. However, completely satisfactory answers for metastasis onset have not yet been found. Due to the increased prevalence of neoplasia and to the existence of various cellular tumor lines resistant to cytotoxic therapy, the research of new active agents is justified [18, 19].

The current study is aimed at testing a newly synthetized heterocyclic SB in terms of antimicrobial activity against Gram-positive and Gram-negative bacteria and antifungal effects against *Candida* strains [20, 21], as well as to evaluate the biocompatibility of the SB *in vitro* on human endothelial cells and the ability of this SB to modulate oxidative stress, by assessing enzymes involved in cellular antioxidant defense.

## 2. Materials and Methods

### 2.1. Synthesis of the Schiff Base

All reagents and solvents used were purchased from Sigma-Aldrich and were used without further purification. The starting compound was previously reported and was synthesized by us according to methodologies described in the literature [21].

**Scheme 1:**
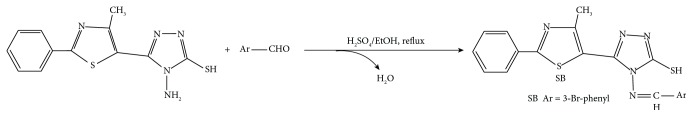
Synthesis of the Schiff base (SB).

The synthesis of Schiff base (SB) 4-(3-bromobenzylideneamino)-5-(4-methyl-2-phenylthiazol-5-yl)-4H-1,2,4-triazole-3-thiol was made using a general procedure (Scheme 1) [21]. 2 mmol (0.578 g) of 4-amino-5-(4-methyl-2-phenylthiazol-5-yl)-4H-1,2,4-triazole-3-thiol was suspended in 10 mL of absolute ethanol. The resulting suspension was added with an alcoholic solution of 2 mmol of 3-bromobenzaldehyde in 5 mL of absolute ethanol and 2-3 drops of concentrated H_2_SO_4_, as a catalyst. The reaction mixture was refluxed for 6 h. The obtained precipitate was filtered hot and washed with absolute ethanol, and then, it was dried and recrystallized from dimethyl sulfoxide (DMSO).

### 2.2. In Vitro Antibacterial and Antifungal Screening

#### 2.2.1. Preparation of Sample Solution

SB was dissolved in DMSO, at a final concentration of 100 *μ*g/mL. Sample solution was stored at 4°C [22, 23].

#### 2.2.2. Inhibition Zone Diameter Measurements

Antimicrobial activity was tested *in vitro* using the agar disk diffusion method through the measurement of the inhibition zone diameters. Agar plates were inoculated with a standardized inoculum of the test microorganisms: two Gram-negative bacterial strains—*Salmonella enteritidis* ATCC 14028 and *Escherichia coli* ATCC 25922, two Gram-positive bacterial strains—*Listeria monocytogenes* ATCC 19115 and *Staphylococcus aureus* ATCC 49444, and a fungal strain—*Candida albicans* ATCC 10231. Petri plates with Mueller Hinton Agar (20.0 mL) were used for all bacterial tests. Mueller-Hinton medium supplemented with 2% glucose (providing adequate growth of yeasts) and 0.5 g/L methylene blue (providing a better definition of the inhibition zone diameter) was used for antifungal testing. Each paper disk was impregnated with 10 *μ*L of solution (100 *μ*g compound/disk). The filter paper disks were placed on Petri dishes previously seeded “in layer” with the tested bacterial strain inoculums. Then, Petri dishes were maintained at room temperature to ensure the equal diffusion of the compound in the medium, and afterwards, the dishes were incubated at 37°C for 24 hours. Inhibition zones were measured after 24 hours of incubation. Assessment of the antimicrobial effect was realized by measuring the diameter of the growth inhibition zone. Ciprofloxacin (10 *μ*g/well) and fluconazole (25 *μ*g/well) were used as *standard* antibacterial and antifungal *drugs.* DMSO was used for comparison, as a negative control, for all experiments, and it did not inhibit the growth of microorganisms (diameter = 6 mm). The clear halos with a diameter larger than 10 mm were considered positive results [22, 23]. Tests were performed in triplicate, and values are presented as the average value ± standard deviation.

#### 2.2.3. Determination of Minimum Inhibitory Concentrations (MICs), Minimum Bactericidal Concentrations (MBCs), and Minimum Fungicidal Concentrations (MFCs)

Minimum inhibitory concentrations (MICs), minimum bactericidal concentrations (MBCs), and minimum fungicidal concentrations (MFCs) were determined by an agar dilution method. Strains of microorganisms used were as follows: *Salmonella enteritidis* ATCC 14028, *Escherichia coli* ATCC 25922, *Listeria monocytogenes* ATCC 19115, *Staphylococcus aureus* ATCC 49444, *Candida albicans* ATCC 10231, *Candida albicans* (ATCC 18804), and *Candida krusei* (ATCC 6258) [22–26]. For the experiment, 100 *μ*L nutrient broths were placed in a 96-well plate, and sample solution at high concentration (100 *μ*g/mL) was added into the first rows of the microplates. 10 *μ*L of culture suspensions was inoculated into all the wells. The plates were incubated at 37°C for 16-24 hours (48 hours for fungi). The reference drugs, ciprofloxacin and fluconazole, were used in the same concentrations.

### 2.3. Determination of Antioxidant Activity by DPPH (2,2-Diphenyl-1-picrylhydrazyl) Bleaching Assay

The DPPH antioxidant activity assay was done as previously described, with minor modification. SB was dissolved in DMSO (1 mg/mL). DPPH∙ radical was dissolved in methanol (0.25 mM). Equal volumes (1.0 mL) of methanolic DPPH solution and sample solution (or standard) in methanol at different concentrations have been used. The mixtures were incubated for 30 min at 40°C in a thermostatic bath; absorbance was measured at 517 nm. The percent DPPH scavenging ability was calculated as follows: DPPH scavenging ability = (*A*_control_ − *A*_sample_)/*A*_control_ × 100, where *A*_control_ is the absorbance of DPPH radical and methanol (containing all reagents, except the sample) and *A*_sample_ is the absorbance of the mixture of DPPH radical and sample. A curve of % DPPH scavenging ability versus concentration was plotted, and IC_50_ values were calculated. The IC_50_ value is the sample concentration required to scavenge 50% of DPPH free radicals. The lesser the IC_50_ value, the stronger the antioxidant capacity. Thus, if IC_50_ ≤ 50 *μ*g/mL, the sample shows a high antioxidant capacity; if 50 *μ*g/mL < IC_50_ ≤ 200 *μ*g/mL, the sample has a moderate antioxidant capacity; if IC_50_ > 200 *μ*g/mL, the sample has poor or no activity. BHT (butylated hydroxytoluene) and trolox (6-hydroxy-2,5,7,8-tetramethylchroman-2-carboxylic acid) were used as positive controls [20, 27–30].

### 2.4. Assessment of the Ability of the SB to Modulate Inflammatory Response and Oxidative Stress on Cell Cultures

#### 2.4.1. Cell Source

Human umbilical vein endothelial cells (HUVECs, Promocell, Hamburg, Germany) were used. The cells were grown in RPMI medium supplemented with 5% fetal calf serum, 50 *μ*g/mL gentamycin, and 5 ng/mL amphotericin (Biochrom Ag, Berlin, Germany). Cell cultures in the 13^rd^ to 15^th^ passages were used. SB was diluted in DMSO (Biochrom Ag, Berlin, Germany) to obtain a stock solution of 1 mg/mL. The stock solution was used to make further dilutions in complete cell growth medium, immediately prior to the experiments. The final DMSO concentration was lower than 0.05%, a nontoxic concentration for the cells [31].

#### 2.4.2. Cell Viability Testing

Cells cultured at a density of 10^4^/well on ELISA 96-well plaques (TPP, Switzerland) were settled for 24 hours, then exposed to different concentrations of the substance ranging from 0.001 to 200 *μ*g/mL. Viability was measured by the colorimetric measurement of a colored compound—formazan, generated by viable cells using the CellTiter 96® AQueous Non-Radioactive Cell Proliferation Assay (Promega Corporation, Madison, USA). Readings were done at 540 nm, using an ELISA plate reader (Tecan, Mannedorf, Austria). Results were presented as OD_540_. All experiments were performed in triplicate. Untreated cultures were used as controls [32].

#### 2.4.3. Experimental Design

Four groups were made: (1) control cells treated only with medium, (2) cells exposed to a high-glucose (4.5 g/L) medium, (3) cells treated with SB 0.001 *μ*g/mL, and (4) cells concomitantly exposed to high glucose and SB (0.001 *μ*g/mL). All groups were treated for 24 hours. Afterwards, cells were used for the assessment of cytoskeleton modifications—phalloidin staining (fluorescence microscopy), oxidative stress (Western blot measurement of SOD1, COX2, and inducible NOS2 and spectrophotometric measurement of MDA), and inflammation (ELISA measurement of TNF-*α*).

#### 2.4.4. Cell Lysis

The cell lysates used in the following experiments were prepared as previously described [33]. Protein concentrations were determined by the Bradford method, according to the manufacturer's specifications (Bio-Rad, Hercules, California, USA) and using bovine serum albumin as standard. For all assays, the lysates were corrected by total protein concentration.

#### 2.4.5. Oxidative Stress and Inflammation Assessment

Quantification of malondialdehyde (MDA) a marker for the peroxidation of membrane lipids was performed by spectrophotometry, as previously described [34]. All reagents were purchased from Sigma-Aldrich. Data were expressed as nM/mg protein [35]. Following viability testing and following the assessment of the MDA level, cells used in further experiments were treated with a concentration of 0.001 *μ*g/mL.

TNF-*α* ELISA Immunoassay kit from R&D Systems, Inc. (Minneapolis, USA) was used. Cell supernatants were treated according to the manufacturer's instructions; readings were done at 450 nm with correction wavelength set at 540 nm, using an ELISA plate reader (Tecan) [33].

Lysates (20 *μ*g protein/lane) were separated by electrophoresis on SDS PAGE gels and transferred to polyvinylidene difluoride membranes, using a Bio-Rad Miniprotean system (Bio-Rad). Blots were blocked and then incubated with antibodies against superoxide dismutase 1 (SOD1), cyclooxygenase 2 (COX2), and inducible nitric oxide synthase 2 (NOS2), then further washed and incubated with corresponding secondary peroxidase-linked antibodies. All antibodies were acquired from Santa Cruz Biotechnology. Proteins were detected using Supersignal West Femto Chemiluminescent substrate (Thermo Fisher Scientific, Rockford IL, USA) and a Gel Doc Imaging system equipped with a XRS camera and Quantity One analysis software (Bio-Rad). Glyceraldehyde 3-phosphate dehydrogenase (GAPDH, Trevigen Biotechnology, Gaithersburg, MD (Maryland), USA) was used as a protein loading control.

Phalloidin-FITC 50 *μ*g/mL (Sigma-Aldrich, St. Louis, MO, USA) a marker for actin myofilaments (green) was used, according to the manufacturer's instructions. Cells were seeded in chamber slides at a density of 5 × 10^4^/chamber, allowed to settle for 24 hours, and then exposed to high glucose and SB as described above. Treated cells were then stained with phalloidin-FITC. Images of cells were documented at a magnification of 20x, using an inverted microscope Olympus BX40 equipped with an Olympus CKX-RFA fluorescent lamp and an E330 camera (Olympus, Hamburg, Germany).

### 2.5. Statistical Analysis

The statistical significance of the differences between the control group and the treated groups was assessed with the nonparametric Kruskal-Wallis test for multiple groups, followed by a post hoc analysis using the Conover test. Correlation coefficients between parameters have been calculated using Spearman's correlation coefficient for ranks (rho). Statistical tests were performed using MedCalc version 18.11.3 and GraphPad Prism Software version 8.0.2. The results were considered statistical significant at *p* < 0.05.

## 3. Results

### 3.1. Chemical Characterization of the SB

The SB structure was confirmed by elemental analysis and on the basis of its mass spectrum (MS), infrared spectrum (IR), and nuclear magnetic resonance (^1^H NMR and ^13^C NMR) spectra [21].

*4-(3-Bromobenzylideneamino)-5-(4-methyl-2-phenylthiazol-5-yl)-4H-1,2,4-triazole-3-thiol*. Yield 80.3% (0.366 g); m.p. 268-270°C; light yellow powder; Anal. Calcd for C_19_H_14_BrN_5_S_2_ (456.38): C, 49.89; H, 3.06; N, 15.33; S, 14.02; Found: C, 50.1; H, 3.07; N, 15.33; S, 14.07; IR (ATR, cm^−1^): 3104 (*ν* NH_triazole_), 1618 (*ν* -N=CH-), 1274 (*ν* C=S); 1055 (*ν* C-Br); ^1^H NMR (500 MHz, DMSO-*d*_6_, *δ*/ppm): 14.18 (s, 1H, NH), 9.52 (s, 1H, -N=CH-), 7.97–8.06 (d, 2H, ArH), 7.92 (s, 1H, ArH), 7.77 (d, 1H, ArH), 7.59 (d, 1H, ArH), 7.47-7.54 (m, 4H, ArH), 2.41 (s, 3H, CH_3_); ^13^C NMR (125 MHz, DMSO-*d*_6_, *δ*/ppm): 170.12 (C=S), 159.15 (C), 157.66 (CH=N), 153.81 (C), 151.07 (C), 143.96 (C), 135.16 (C), 134.51 (C), 131.21 (CH), 130.93 (2CH), 130.29 (CH), 129.29 (2CH), 128.94 (CH), 128.68 (C), 127.36 (CH), 127.14 (CH), 15.92 (CH_3_); MS (EI, 70 eV) *m*/*z* (%): 457 (M + 1).

### 3.2. Antimicrobial Activity

Results obtained by measuring the diameters of growth inhibition zones of the tested microorganisms, compared to ciprofloxacin and fluconazole, used as standard reference drugs, are presented in Table 1.

MIC, MBC, and MFC values of the new compound are presented in Tables 2 and 3. The results showed that MIC values ranged from 1.95 (*Listeria monocytogenes*) to 62.5 *μ*g/mL, MBC values were between 3.9 and 125 *μ*g/mL, and MFC scores ranged between 62.5 and 125 *μ*g/mL.

### 3.3. In Vitro Antioxidant Capacity

The antioxidant capacity of the SB was determined by the DPPH bleaching method, and BHT and trolox were used as positive controls. The results are displayed in Table 4. The new compound showed a very low IC_50_ value (16.10 *μ*g/mL), similar to that of BHT (16.39 *μ*g/mL).

### 3.4. Cell Viability

**Figure 1:**
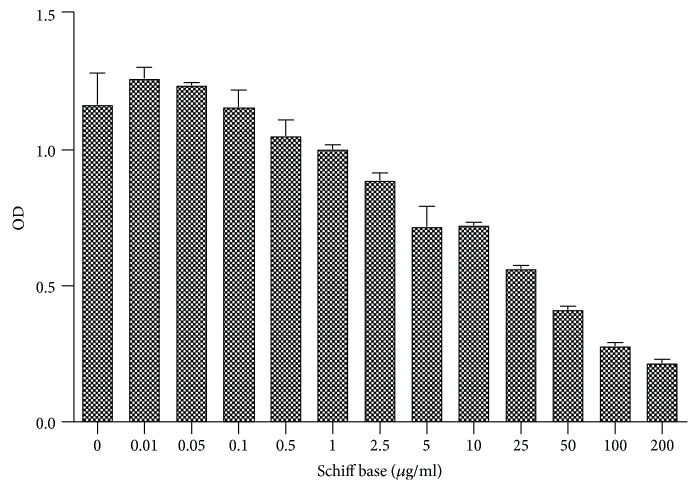
Cell viability testing. Schiff base (SB) was tested for multiple concentrations (0.01-200 *μ*g/mL). Cell viability is presented as OD 540 nm (mean values ± standard deviation at 540 nm, *n* = 3).

SB did not lead to significant changes in HUVEC viability for doses lower than 0.1 *μ*g/mL (Figure 1). Higher concentrations led to a dose-dependent viability decrease, compared with control.

### 3.5. Assessment of the Ability of the SB to Modulate Inflammatory Response and Oxidative Stress on HUVECs

Lipid peroxidation level (MDA), the ability to modulate inflammatory response (TNF-*α*, COX2), and the activity of enzymes involved in the prooxidant/antioxidant equilibrium (SOD1, NOS2) were appreciated. The ability of the SB to modulate oxidative stress was tested *in vitro* on HUVECs, using a glucose-enriched medium [36–38]. A SB concentration of 0.001 *μ*g/mL was used for all experiments.

**Figure 2:**
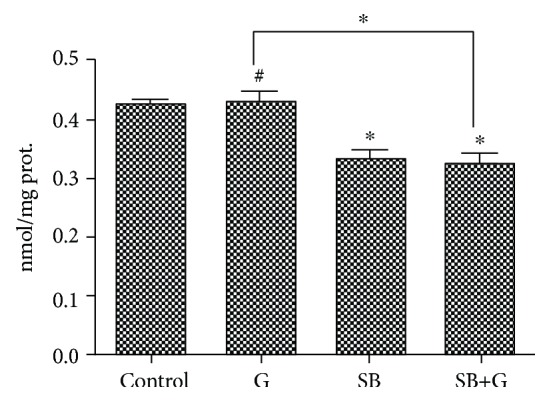
Lipid peroxidation levels (MDA) in endothelial cells exposed to medium (control), glucose (G), Schiff base (SB), and combination treatment (SB+G). Each bar represents the mean ± standard deviation (*n* = 3). ^#^Not significant. ^∗^*p* < 0.05.

The effect of the newly synthetized compound on lipid peroxidation (MDA level) was assessed. SB administration decreased the MDA level compared with both control and glucose-enriched medium, thus reducing the lipid peroxidation in endothelial cells (Figure 2).

**Figure 3:**
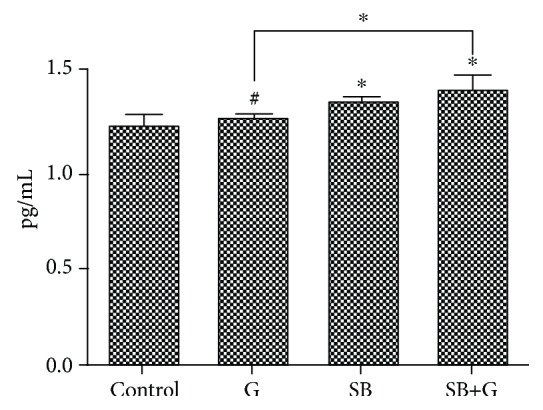
TNF-*α* levels in endothelial cells exposed to medium (control), glucose (G), Schiff base (SB), and combination treatment (SB+G). Each bar represents the mean ± standard deviation (*n* = 3). ^#^Not significant. ^∗^*p* < 0.05.

The TNF-*α* level was quantified through ELISA for the same SB concentration (Figure 3). Glucose-enriched medium slightly increased the TNF-*α* level. SB also increased the TNF-*α* level both alone and in combination with glucose.

The same SB concentration (0.001 *μ*g/mL) was used to further test its effect on the protein level of the enzymes involved in the oxidant/antioxidant equilibrium and in the inflammatory response (SOD1, NOS2, and COX2).

**Figure 4:**
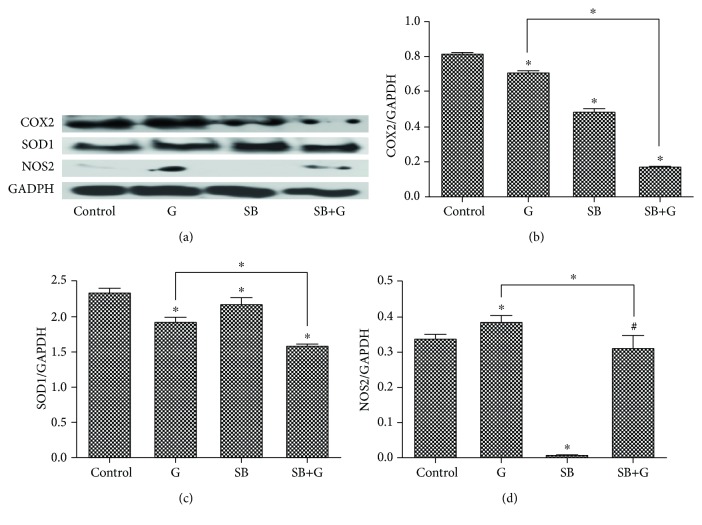
Protein levels of COX2, SOD1, and NOS2 in endothelial cells exposed to medium (control), glucose (G), Schiff base (SB), and combination treatment (SB+G). Comparative Western blot images showing expressions of COX2, SOD1, and NOS2 in HUVECs (b, c, d). Image analysis of Western blot bands (a) was performed by densitometry; results were normalized to GAPDH. Each bar represents the mean ± standard deviation (*n* = 3). ^#^Not significant. ^∗^*p* < 0.05.

An inflammatory marker (COX2) and antioxidant enzyme (constitutive SOD1 and inducible NOS2) expression was quantified by Western Blot (Figure 4).

COX2, an inflammatory marker, significantly decreased after both glucose and SB treatments, compared to control. Combined exposure (SB+G) strongly decreased the protein level of COX2 (Figure 4(b)). This finding is consistent with MDA levels and may be due to the antioxidant effect of the SB in this experimental setting. Interestingly, it is not consistent with TNF-*α*, a fact which might be explained by a different mechanism than oxidative stress that triggers an increase of TNF-*α*. Exposure to glucose-enriched medium significantly decreased SOD1. SB slightly decreased SOD1 activity compared with the control group, but SOD1 activity was maintained at a significantly higher level, compared with glucose (*p* < 0.05). Combination (SB+G) treatment significantly decreased SOD1 compared with both glucose and control (Figure 4(c)). Exposure to glucose increased significantly NOS2. The SB drastically decreased the NOS2 level compared with both the control and glucose groups (Figure 4(d)).

Correlation analysis, using Spearman's coefficient for rank correlation (Table 5), revealed statistically significant positive correlations between MDA and enzyme (COX2, SOD1, and NOS2) levels. On the other hand, the TNF-*α* level negatively correlates with both MDA and all enzymes measured.

**Figure 5:**
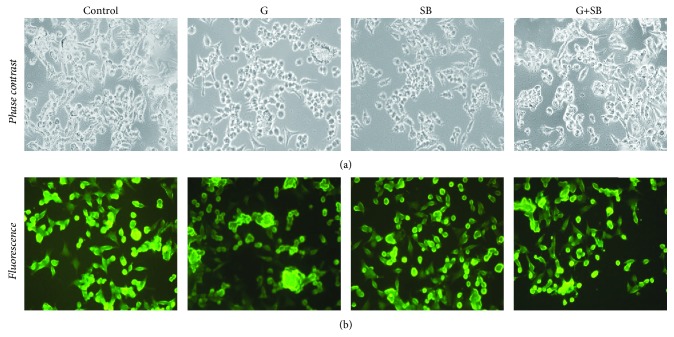
Images of HUVECs treated with medium (control), glucose (G), Schiff base (SB), and combination treatment (SB+G), stained with phalloidin-FITC; the same microscopic field is presented as phase contrast images (a) and fluorescence images (b) for comparison (pictures taken through an Olympus BX inverted microscope, original magnification 20x).

Cell morphology does not seem to be affected by exposure to the Schiff base compared to control. When exposed to high-glucose concentration, cells had a tendency to conglomerate and to form multilayered spherical bodies, with alteration of the actin filament disposition. The aspect of the cells receiving combination treatment was similar to those of controls (Figure 5).

## 4. Discussion

The structure of the Schiff base was established by elemental analysis and on the basis of its mass spectrum (MS), infrared spectrum (IR), and nuclear magnetic resonance (^1^H-NMR and ^13^C-NMR) spectra. The results of the C, H, N, S quantitative elemental analysis were in agreement with the calculated values, within ±0.4% of the theoretical values. The spectral data confirmed the formation of the SB. The recorded mass spectrum revealed the correct molecular ion peak (*M* + 1), as suggested by the molecular formula. The absence of the NH_2_ asymmetric and symmetric stretching vibrations at 3281 cm^−1^ and 3186 cm^−1^, and the presence of N=CH stretch absorption bands at 1618 cm^−1^ in the IR spectrum of the final compound provided strong evidences for the formation of the SB. The ^1^H-NMR spectrum of the starting compound was recorded a signal characteristic for the amino protons, as a singlet, at 5.73 ppm. The absence of this signal from the ^1^H-NMR spectrum of the newly synthesized compound and the presence of a singlet characteristic to the N=CH proton at 9.52 ppm further confirmed the condensation between the 4-amino-5-(4-methyl-2-phenylthiazol-5-yl)-4*H*-1,2,4-triazole-3-thiol and the 3-bromo-phenyl-carbaldehyde. The ^13^C-NMR spectrum of the newly synthesized compound was consistent with the proposed structure.

The aim of the present study was to evaluate the antibacterial and antifungal activity of a new SB as well as its ability to modulate oxidative stress.

The new thiazolyl SB exerted moderate to good antibacterial activity against tested strains (Tables 1–3). The inhibition of bacterial growth was more pronounced in Gram-negative bacteria, especially in *Pseudomonas aeruginosa* strain, where the SB showed better activity compared with ciprofloxacin, used as the reference drug. Regarding antifungal activity, the compound showed a better anti-*Candida* effect than fluconazole, used as the reference drug. Previous studies showed that SBs have the ability to modulate oxidative stress [17, 39]. This ability can be exploited in order to use them as antibacterial drugs and/or as potential oxidative stress modulators in medicine. The SB was tested on endothelial cells exposed to a glucose-enriched environment.

High-carbohydrate intake, impaired glucose tolerance, and diabetes mellitus lead to hyperglycemia and chronic inflammatory status. Endothelial lesions are often involved in the pathology of these conditions [40]. During inflammatory episodes, such as response to injury, nitric oxide (NO) is released in order to modulate vascular tone. Since glycocalyx plays an important role in transducing the fluid stress to the cytoskeleton of the endothelial cells, vasodilator substance production is stimulated [40–42]. High-glucose concentration increases oxidative stress and influences the structure of the cytoskeleton. Exposure to high-glucose hyperosmolar medium induces, using an AQP1-dependent mechanism, remodeling of the F-actin and cytoskeleton [43]. Our results are consistent with these findings (Figure 5). A high-glucose level led to mitochondrial dysfunction and increased production of ROS [44, 45].

A glucose-enriched environment also triggers the release of proinflammatory cytokines, such as tumor necrosis factor alpha (TNF-*α*), by the cells involved in immune reactions [46, 47], along with other proinflammatory molecules, such as CRP, interleukin 6, intercellular adhesion molecule 1, and VCAM-1. In diabetic patients, TNF-*α* was related with an atherogenic profile and with vascular complications [48]. A similar effect was obtained in our study, where higher levels of TNF-*α* were observed after hyperglycemia exposure. This effect was also seen after SB treatment and was augmented by the combined SB and high-glucose concentration. However, TNF-*α* production was negatively correlated with MDA and antioxidant enzymes (Table 5). This suggests that the increased TNF-*α* was not produced through enhanced oxidative stress, but through a different mechanism. Its clarification requires further studies. Since TNF-*α* acts as a promoter of leucocyte adhesion to the endothelium, the SB might be beneficial as antimicrobial, local immune response, and oxidative status modulator in the treatment of infectious diseases.

The results obtained by the DPPH study showed that the SB exhibited antioxidant activity. The low IC_50_ value, similar to the positive control (BHT), reflects a strong antioxidant activity *in vitro*. The new compound showed radical scavenging activity according to the DPPH method, the presence of the -SH group being probably responsible of the radical scavenging activity [49–51]. The effect of the SB on the oxidative stress was also tested *in vitro* on cell cultures (HUVECs), by assessing the MDA level, a marker of lipid peroxidation and the expression of two enzymes involved in the oxidative equilibrium (SOD1 and NOS2). The results showed that, at the tested concentration (0.001 *μ*g/mL), SB decreased lipid peroxidation (MDA) and the protein level of certain enzymes involved in the modulation of oxidative stress and inflammatory response (COX2 and NOS2). These changes are consistent with the DPPH result and suggest an anti-inflammatory effect of the tested SB, mostly by interfering with the prooxidant mediators.

The ability of the SB, in low concentrations, to decrease lipid peroxidation, might be explained by its capacity to form complexes with the bivalent and trivalent metal ions located in the active center of the enzymes involved in the onset of the oxidative stress or in the scavenging of the prooxidant molecules [52–57]. The antioxidant effect on the human cells (Figures 2 and 4) is also consistent with the absence of morphological changes of the cells observed in the present study (Figure 5).

Considering antibacterial activity, especially against *Pseudomonas aeruginosa*, the decrease of the NOS2 protein level in HUVECs after SB exposure, it might be possible that the synthesis of NO by bacteria could also be reduced. One of the many proposed roles of NO in bacteria is to help protect the bacteria from host cell antibiotic-induced oxidative stress; therefore, the inhibition of bacterial nitric oxide synthase has been identified as a promising antibacterial strategy, especially for resistant bacteria [58].

Nitric oxide synthase (NOS) inhibitor NO-donating drugs were reported to inhibit IL-1*β* production, modulate PGE_2_ production, and protect against apoptosis in human endothelial cells and human monocytes [59]. In type 2 diabetes, hyperglycemia stimulates endothelial cell migration in the retina, leading to retina neoangiogenesis and visual impairment by CXC receptor-4 stimulation and activation of the PI3K/Akt/eNOS signaling pathway. Therefore, SB modulation of the NOS2 might be beneficial for the endothelial dysfunction in hyperglycemia [60, 61].

Recent studies showed that the antibacterial and antifungal activity in general and antibiofilm activity of some newly identified classes seem to correlate with their ability to induce ROS synthesis [5]. The SB showed an anti-*Candida* effect, with a twofold increased activity compared with the consecrated antifungal fluconazole (Tables 2 and 3). Also, the results showed that SB reduced the SOD1 level and increased the activity of the proinflammatory cytokine (TNF-*α*). The antifungal effect could also be explained by the ability of the tested SB to form complexes between the azomethine group and the metal from the active center of the enzymes and also by its capacity to induce ROS production, similar with some antifungal azoles (e.g., miconazole) [5].

Additional studies are needed in order to clarify the effect of such compounds as SB and their role as adjuvant antioxidant, antimicrobial, and local immune response modulators (TNF-*α*) in the treatment of infectious diseases.

## 5. Conclusions

The new Schiff base exhibited antibacterial effects on both Gram-positive and Gram-negative bacteria, as well as antifungal activity against *Candida albicans*. The results of the present study show that the new SB plays a role in the prooxidant/antioxidant equilibrium. In the tested dose, SB does not change endothelial cell morphology, has an antioxidant effect, as demonstrated by the DPPH test, decreased lipid peroxidation (MDA), and decreased the inducible NOS2 level. Therefore, it can be considered a potential candidate with promising antioxidant properties that may be used as an adjuvant therapy in diseases caused by excessive free radical production. The decrease in COX2 and NOS2 levels also might suggest an anti-inflammatory action. A possible mechanism for the antibacterial activity on Gram-negative bacilli could include the decrease of the bacterial NOS level and the formation of complexes with metals located in the active center of certain bacterial enzymes. Also, the SB might potentially act as an antifungal agent, through ROS production in fungal biofilm cells. Its clarification requires further studies.

## Figures and Tables

**Table 1:** Inhibition zone diameters on tested microorganisms.

Samples	Diameter of the inhibition zone (mm)				
	SA	LM	EC	ST	CA
*Schiff base*	14	14	14	18	18
Ciprofloxacin	28	18	27	22	—
Fluconazole	—	—	—	—	25

SA: *Staphylococcus aureus*; LM: *Listeria monocytogenes*; EC: *Escherichia coli*; ST: *Salmonella typhimurium*; CA: *Candida albicans.*

**Table 2:** Minimum inhibitory concentrations (MIC).

Samples	Minimum inhibitory concentration (MIC (*μ*g/mL))					
	SA	LM	PA	ST	CA (ATCC 10231)	CA (ATCC 18804)
*Schiff base*	31.25	1.95	1.95	62.5	62.5	31.25
Ciprofloxacin	1.95	3.9	3.9	0.97	—	—
Fluconazole	—	—	—	—	62.5	62.5

SA: *Staphylococcus aureus*; LM: *Listeria monocytogenes*; PA: *Pseudomonas aeruginosa*; ST: *Salmonella typhimurium*; CA: *Candida albicans*.

**Table 3:** Minimum bactericidal (MBC) and minimum fungicidal concentrations (MFC).

Samples	MBC (*μ*g/mL)				MFC (*μ*g/mL)		
	SA	LM	PA	ST	CA (ATCC 10231)	CA (ATCC 18804)	CK (ATCC 6258)
*Schiff base*	62.5	3.9	3.9	125	125	62.5	62.5
Ciprofloxacin	3.9	7.8	7.8	1.95	—	—	—
Fluconazole	—	—	—	—	125	125	125

SA: *Staphylococcus aureus*; LM: *Listeria monocytogenes*; PA: *Pseudomonas aeruginosa*; ST: *Salmonella typhimurium*; CA: *Candida albicans*; CK: *Candida krusei.*

**Table 4:** Antioxidant capacity using the DPPH method.

Samples	IC_50_ (*μ*g/mL)
*Schiff base*	16.10 ± 1.2
BHT	16.39 ± 0.9
Trolox	11.98 ± 0.4

**Table 5:** Spearman's coefficient of rank correlation (rho) between the oxidative stress and inflammation markers in HUVECs.

	MDA	TNF-*α*	COX2	SOD1	NOS2
MDA	1.00	-0.776^∗∗^	0.699^∗^	0.462	0.595^∗^
TNF-*α*		1.00	-0.818^∗∗^	-0.566	-0.455
COX2			1.00	0.755^∗∗^	0.431
SOD1				1.00	-0.144
NOS2					1.00

^∗^ *p* < 0.05, ^∗∗^*p* < 0.01.

## Data Availability

The data used to support the findings of this study are included within the article.
